# Performance evaluation of different empirical models for reference evapotranspiration estimation over Udhagamandalm, The Nilgiris, India

**DOI:** 10.1038/s41598-024-60952-4

**Published:** 2024-05-30

**Authors:** P. Raja, Fathima Sona, U. Surendran, C. V. Srinivas, K. Kannan, M. Madhu, P. Mahesh, S. K. Annepu, M. Ahmed, K. Chandrasekar, A. R. Suguna, V. Kumar, M. Jagadesh

**Affiliations:** 1 ICAR-Indian Institute of Soil and Water Conservation (IISWC), Research Centre, Ooty, India; 2 ICAR-Indian Institute of Soil and Water Conservation, Research Centre, Koraput, Odisha India; 3 Centre for Water Resources Development and Management, Kozhikode, 673571 Kerala India; 4 Environmental Assessment Division, Indra Gandhi Centre for Atomic Research, a CI of Homi Bhabha National Institute, Kalpakkam, Tamil Nadu India; 5 ICAR-Indian Institute of Soil and Water Conservation (IISWC), Research Centre, Dehra Dun, India; 6 National Remote Sensing Centre (NRSC), Indian Space Research Organization (ISRO), Hyderabad, India; 7 Regional Remote Sensing Centre - ISRO, Bangalore, Karnataka India; 8 Department of Agricultural Engineering, ACRI, TNAU, Madurai, India; 9 Department of Soil Science and Agricultural Chemistry, Tamil Nadu Agricultural University (TNAU), Coimbatore, Tamil Nadu India; 10 Present Address: ICAR-Sugarcane Breeding Institute, Coimbatore, Tamil Nadu India

**Keywords:** Evapotranspiration, FAO-PM method, Empirical models, Climate sciences, Hydrology

## Abstract

Evapotranspiration (ET_o_) is an important component of the hydrological cycle and reliable estimates of ET_o_ are essential for assessing crop water requirements and irrigation management. Direct measurement of evapotranspiration is both costly and involves complex and intricate procedures. Hence, empirical models are commonly utilized to estimate ET_o_ using accessible meteorological data. Given that empirical methods operate on various assumptions, it is essential to assess their performance to pinpoint the most suitable methods for ET_o_ calculation based on the availability of input data and the specific climatic conditions of a region. This study aims to evaluate different empirical methods of ET_o_ in the tropical highland Udhagamandalam region of Tamil Nadu, India, utilizing sixty years of meteorological data from 1960–2020. In this study, 8 temperature-based and 10 radiation-based empirical models are evaluated against ET_o_ estimates derived from pan evaporation observation and the FAO Penman–Monteith method (FAO-PM), respectively. Statistical error metrics indicate that both temperature and radiation-based models perform better for the Udhagamandalam region. However, radiation-based models performed better than the temperature based models. This is possibly due to the high humidity of the study region throughout the year. The results suggest that simple temperature and radiation-based models using minimum meteorological information are adequate to estimate ET_o_ and thus find potential application in agricultural water practices, hydrological processes, and irrigation management.

## Introduction

Evapotranspiration is an important component of the hydrologic cycle in the land–atmosphere system because it accounts for a sizable portion of the moisture lost from a plant canopy. Evapotranspiration (ET) plays a vital role in both the global hydrological cycle and regional water budgets. It encompasses the combined processes of surface water evaporation, bare soil evaporation, and plant transpiration, representing the movement of water from the Earth's surface and soil to the atmosphere as vapor. This dynamic process influences the distribution and availability of water resources on both at a local and global scale and this has gained utmost importance due to the change in climate^1,2^. Hence calculating reference crop evapotranspiration is crucial for determining crop water needs and for scheduling irrigation^3^. Reference evapotranspiration (ET_o_) quantifies the maximum amount of moisture that is lost to the atmosphere through the combined process of evaporation and plant transpiration for a given atmospheric condition. The goal of determining evapotranspiration is to exclude crop-specific variations from the evapotranspiration process by assuming constant crop conditions. However, the reference crop is not properly defined in this specification, which may pose a challenge in the total elimination of crop components. Several methods are developed for estimation of ET_o_ using direct and indirect approaches. Among these methods, the lysimeter technique is often regarded as the most reliable for estimating crop evapotranspiration (ETc), providing detailed insights into soil water retention and excess irrigation percolation simultaneously. However, the significant expenses and the labor-intensive nature of lysimeter installation and expensive maintenance hinder their widespread adoption in agricultural systems. This limitation restricts the use of lysimeter in the areas within a research site where measurements can be conducted, thereby constraining the spatial coverage of evapotranspiration assessments^4^. The other methods for estimation of evaporation include pan evaporimeter, water budgeting, empirical models, etc. Among these, the different empirical methods of ET_o_ estimation can be grouped into empirical formulations based on radiation, temperature, mass transfer, and combination theory types. Over time, a variety of empirical methods have been developed to calculate evapotranspiration, but none of them can be deemed flawless due to the wide variations in climatic conditions in different parts of the world. To overcome the ambiguity, the American Society of Civil Engineers (ASCE) and Food and Agriculture Organization (FAO) jointly formulated the Penman–Monteith equation for determining Reference Evapotranspiration by combining all the responsible meteorological parameters /processes^5^.

Although several models or equations have been reported in the literature for the estimation of ET_o_, there is uncertainty about which equations are appropriate for a specific climatic situation, and each model needs exact local calibration^6^. A variety of empirical and semi-empirical approaches have been used in the Indian context for estimating reference evapotranspiration (ET_o_) based on their performance in other areas of the world^7,8^.

Muniandy et al. compared 26 empirical models and pan evaporation calculation techniques at the Modern Agriculture Centre in Kluang, Johor, Peninsular Malaysia^9^. They reported that, when compared to other models, the mass transfer-based Penman model produced the best ET_o_ results. Similar to this, Bourletsikas et al. evaluated ET_o_ using 24 different empirical models^10^. They discovered that to improve performance in Greece, mass transfer-based models must be calibrated. In addition, 14 distinct empirical ET_o_ equations were investigated by Gao et al. in various climate zones of China. It has been shown that in dry, semi-dry, and humid conditions, respectively, the Priestley–Taylor, Hargreaves, and Makkink approaches performed best^11^. In northwest China, Celestin et al. compared the 32 empirical ET_o_ equations with the FAO56-PM using temperature, solar radiation, and mass transfer data^12^. They found that the Mahringer and World Meteorological Organization (WMO) methodologies generated the best results. Djaman et al. reported that the Abtew ET_o_ equation, which is based on solar radiation and a maximum temperature, performed best when compared to the FAO-PM model throughout the three climate zones of Mali^13^. According to Zakeri et al. the Jensen–Haise approach was the most effective empirical equation in the dry Semnan province^14^. The climatic conditions in India are highly diverse over different zones and Mandal et al. suggested that the Hargreaves method provides better ET_o_ estimates for different stations in India^15^.

The applicability of any evapotranspiration model is limited by the availability of input data. Therefore, there is a strong need to identify and understand the most suited empirical models for specific areas to obtain ET_o_ estimates from limited data sets. Various studies attempted to make an approximation to the ET_o_ for different climatic conditions using a limited amount of information^14^. The main objective of this study is to evaluate various empirical approaches (18 models) to identify a suitable model which best fits the climatic conditions of Udhagamandalam in the Nilgiri district of Tamil Nadu by comparing all the 18 empirical models against ET_o_ estimates derived from pan evaporation observation and the FAO Penman–Monteith method (FAO-PM), respectively.

## Materials and methods

### Study area

**Figure 1:**
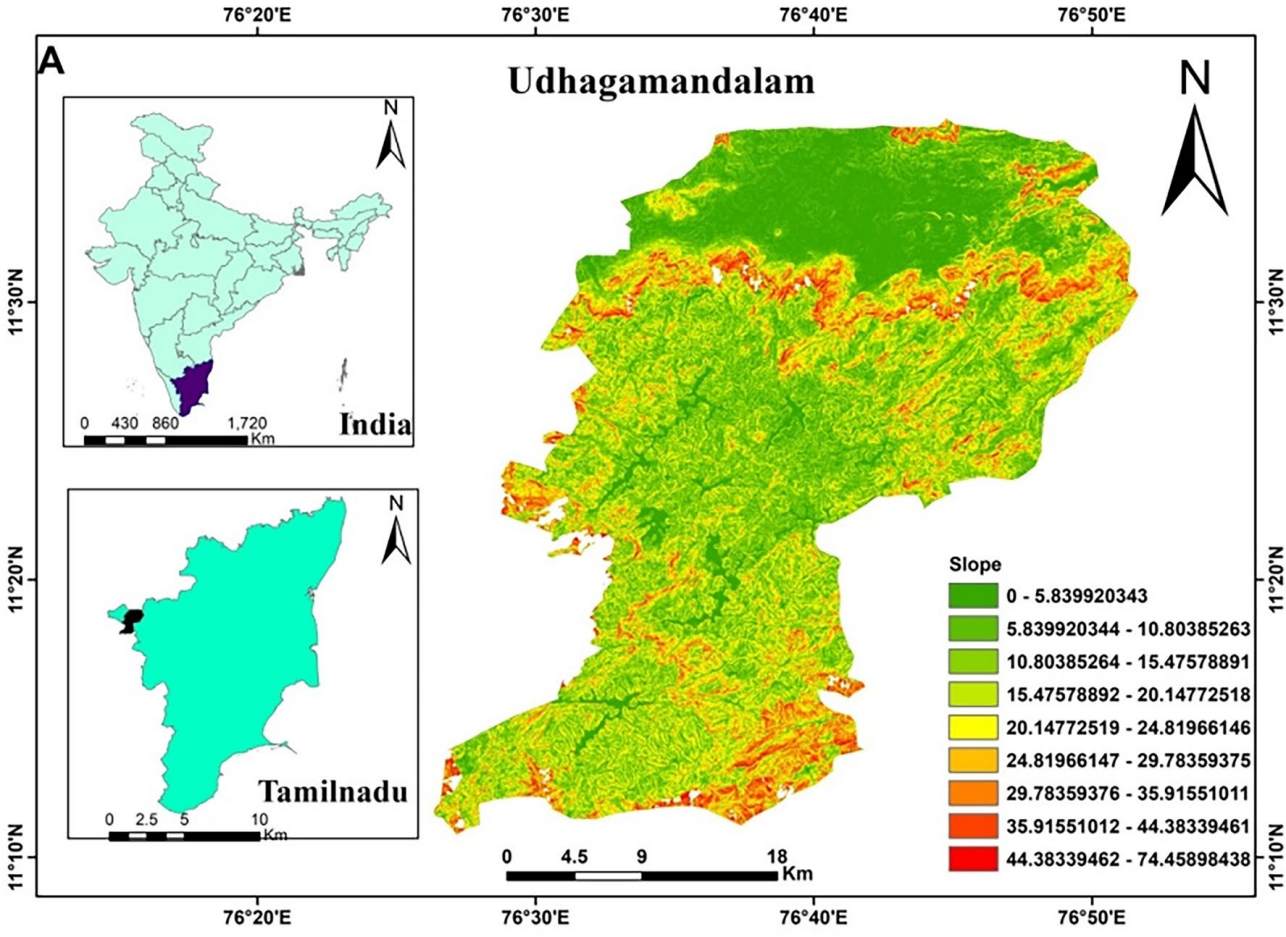
Map of study area. (Figure created by the 3rd author—Using Administrative Boundary of India and its districts using ARC GIS Software Version-10.1-https://www.arcgis.com).

The study area Udhagamandalam is located on Western Ghats between 11°24′36″ N latitude and 76°41′15″ E longitude with an average altitude of 2218 m in the Nilgiri district of Tamil Nadu, India (Fig. 1). Udhagamandalam has a cold climatic topography with a tropical highland climate. Despite located in the tropics, Udhagamandalam experiences pleasant weather year-round, in contrast to most of South India. On the other hand, the nights in January and February are monotonously cold^17^. The average monthly weather parameters over 60 years (1960–2020) are represented in Table 1. The average maximum temperature varies from 17.6 °C in July to 22.1 °C in April, whereas the average minimum temperature ranges from 7.3 °C in January to 12.6 °C in May. The average annual rainfall is 1250 mm and a major amount (74%) of rainfall occurs during the southwest monsoon season (June to September). The data of meteorological parameters like maximum and minimum temperature, rainfall, wind speed, relative humidity, pan evaporation and sunshine hours were collected from the meteorological observatory at the Indian Institute of Soil and Water Conservation, Udhagamandalam. The average daily temperature is obtained by summing the maximum and minimum temperatures during a 24-h period and dividing them by two.

**Table 1:** Average monthly weather parameters of Udhagamandalam.

Month	Maximum temperature (°C)	Minimum temperature (°C)	Rainfall (mm)	Maximum relative humidity %	Minimum relative humidity %	Wind speed (km hr^−1^)	Sunshine hours (hrs)	Pan evaporation (mm)
January	19.5	7.3	1.7	95.0	51.1	4.09	8.5	3.5
February	20.1	8.3	3.0	94.2	51.0	4.18	8.6	3.9
March	21.2	10.3	23.8	93.8	53.4	5.18	8.6	4.3
April	22.1	12.0	69.5	93.1	57.6	4.57	7.7	4.1
May	21.8	12.6	226.0	93.9	63.5	4.54	6.7	3.4
June	18.5	12.0	814.9	95.9	78.2	7.20	5.3	2.5
July	17.6	11.8	753.6	96.2	80.3	8.51	3.0	2.1
August	17.7	11.8	447.6	95.9	78.5	7.59	3.4	2.2
September	18.6	11.5	271.9	95.5	72.1	4.76	4.5	2.5
October	18.5	11.0	295.1	95.6	71.1	3.71	4.8	2.5
November	18.0	9.9	146.2	95.4	65.0	4.22	5.7	2.6
December	18.6	8.5	38.0	95.3	55.4	4.47	7.2	3.0

The study considered 18 empirical models based on 2 categories, i.e., Temperature based and Radiation based. The pan observed reference evapotranspiration which was obtained by multiplying pan evaporation with the pan coefficient value and FAO-PM based reference evapotranspiration are considered as base models for comparison.

1 $${ET}_{o }= {K}_{pan} \times {E}_{pan}$$

where, ET_o_ is the reference crop evapotranspiration (mm/day), Epan is the measured class A pan evaporation (mm/day) and Kpan is the pan coefficient.

Various studies have reported a high correlation between Epan and ET_o_ when evaporation pans are maintained properly^18,19^. Different equations for estimating Kpan values have been proposed by various scholars in recent decades^20–25^. In this study, the Allen and Pruitt^26^ method was used to calculate Kpan value and selection of the Allen and Pruitt method for calculating the Kpan value is justified based on its empirical validation by few of the earlier authors^20–25^, simplicity, adaptability, consideration of local conditions, and its accuracy. These factors make it a reliable and widely accepted approach for estimating pan coefficients and adjusting reference evapotranspiration to local conditions using pan evaporation data.

2a $${K}_{pan}=0.108- \left(3.31 \times {10}^{-4}\times {U}_{2}\right)+\left(0.0422\times {\text{ln}}\left(F\right)\right)+\left(0.1434 \times {\text{ln}}\left(RH\right)\right)-\left[6.31 \times \left({\text{ln}}{\left(F\right)}^{-4}\right)\times {\text{ln}}\left(RH\right)\right]$$

### Empirical methods of ET_o_ calculation

 Several ways are available to measured evapotranspiration using direct and indirect methods. Evapotranspiration can be measured in field condition directly using a weighing lysimeter, pan evaporimeter and water budgeting technique. Even though these methods give accurate data, due to their high cost of installation and maintenance, indirect methods are preferred in most of the conditions. The empirical methods are based on observed meteorological parameters and involve statistical fit coefficients and are generally adequate for a specific climatic or region condition. However, because these methodologies and models were developed for specific climatic zones and have various input data requirements and assumptions, they yield different values^26^. Previous research on numerous scales also revealed that alternative methodologies might produce outcomes that are noticeably different which makes it difficult to choose the right procedure for the intended application^27^.

**Table 2:** Empirical models of ET_o_ calculation.

Model	Equation	References
*Temperature based methods*		
Hargreaves–Samani	ET_o_ = $[0.0023{R}_{a} ({T}_{avg}+17.8)({Tmax- Tmin)}^{0.517}]/\lambda$	Hargreaves and Samani^28^
Schendel	ET_o_ = $16 (\frac{Tmean}{RH})$	Schendel^29^
Kharrufa	ET_o_ = $0.34 \times p\times {T}_{mean}^{1.3}$	Kharrufa^30^
Trajkovic	ET_o_ = $0.0023 {R}_{a }\times {TD}^{0.424} ({T}_{mean}+17.8)$	Trajkovic^31^
Berti	ET_o_ = $[0.00193Ra \left({T}_{mean}+17.8\right) ({Tmax- Tmin)}^{0.5}]/\lambda$	Berti et al.^32^
Blaney-Criddle	ET_o_ = $p\times (\left(0.46\times Tmean\right)+8.13)$	Singh^33^
Papadakis	ET_o_ = $2.5({\text{ema}}-{\text{ea}})$	Papadakis^34^
Ivanov	ET_o_ = $0.00006{(25+{\text{Tmean}})}^{2} (100-{\text{RH}})$	Cunha et al.^35^
*Radiation based methods*		
Makkink	ET_o_ = $0.61\left(\frac{\Delta }{\Delta +\gamma }\right)\left(\frac{{\text{Rs}}}{58.5}\right)-0.12$	Makkink^36^
Jensen–Haise	ET_o_ = $(\frac{{\text{Rs}}}{\uplambda })(0.025{\text{Tmean}}+0.08)$	Jensen and Haise^37^
Irmark-Rs	ET_o_ = $-0.611+0.149 {\text{Rs}}0.079\mathrm{Tmean}$	Irmark et al.^38^
Irmark Rn	ET_o_ = $0.489+0.289 {\text{Rn}}+0.023\mathrm{Tmean}$	Irmark et al.^38^
Caprio	ET_o_ = $\left(\frac{6.1}{{10}^{6}}\right){\text{Rs}} (1.8 {\text{Tmean}}+1.0)$	Caprio^39^
Jones	ET_o_ = ${{{\alpha}}}_{1}(3.87\times {10}^{-3})(\mathrm{Rs }(0.6\mathrm{Tmax}+ 0.4\mathrm{Tmin}+29)$	Jones and Ritchie^40^
Turc	ET_o_ = $0.013 \left(\frac{{\text{Tmean}}}{{\text{Tmean}}+15 }\right)({\text{Rs}}+50)$	Turc^41^
Tabari	ET_o_ = $0.156\mathrm{Rs}-0.0112{\text{Tmax}}+ 0.0733\mathrm{Tmin}-0.478$	Tabari et al.^42^
Priestley–Taylor	ET_o_ = $1.26 \frac{\Delta }{\Delta +\upgamma } \frac{{\text{Rn}}}{\uplambda }$	Priestley and Taylor^43^
Abtew	ET_o_ = $0.53 \frac{{\text{Rs}}}{\uplambda }$	Abtew^44^

Rn = net radiation, MJ m^−2^ day^−1^; Ra = extra-terrestrial radiation, MJ m^−2^ day^−1^; γ = psychrometric constant, kPa °C^−1^; es = saturation vapor pressure, hPa; ea = actual vapor pressure, hPa; in all equations except the Papadakis, Rohwer, Penman, and FAO Penman–Monteith models where es and ea, kPa; Δ = slope of the saturation vapor pressure and temperature curve, kPa °C^−1^; Δ = latent heat of evaporation, MJ kg^−1^; Tmean = average daily air temperature, °C; u = mean daily wind speed at 2 m, m s^−1^; f(u) = function of wind speed; Z = elevation, m; L = local latitude, °(Degree); Td = dew point temperature, °C; Tmin = minimum air temperature, °C; Tmax = maximum air temperature, °C; TD = difference between maximum and minimum temperature, °C; RH = average relative humidity, %; Rs = solar radiation. MJ m^−2^ day^−1^; in all equations except the Turc and Makkink where Rs, Cal m^−2^ day^−1^; and in Caprio model, where Rs, KJm^−2^ day^−1^; ema = saturation vapor pressure at the monthly mean daily maximum temperature, kPa; p = mean annual percentage of daytime hours for different latitudes that can be obtained from Doorenbos and Pruitt^24^; Ld = daytime length in multiples of 12 h. RH_0_sat = saturated vapor density, g m^−3^; Esat = saturated vapor pressure, m bar; KPEC = calibration coefficient i.e. 1.2; α = constant i.e. 1.26; α_1_ = constant i.e. 1.1.

In this study, a total of 18 empirical models are selected which are broadly classified as temperature-based and radiation-based models (Table 2). The temperature-based methods consider maximum, minimum and mean temperatures and relative humidity as major input factors. On the other hand, the radiation-based models consider temperature, solar radiation and net radiation as input parameters. FAO56-Penman–Monteith method is the universally accepted method for the calculation of evapotranspiration over various geographical regions as it is physically based and integrates physiological characteristics and aerodynamic variables^45,46^. It is a combination method based on temperature, radiation, relative humidity, wind speed, and saturation vapor pressure values. The equation is as follows.

2b $${\text{ET}}_{{\text{o}}} = 0.408*\Delta {\text{Rn}} - {\text{G}} + \gamma *\left( {900/\left( {{\text{T}} + 273} \right)} \right)*{\text{U}}2*\left( {{\text{es}} - {\text{ea}}} \right)/\Delta + \gamma *\left( {1 + 0.34*{\text{U}}2} \right)$$

Where ET_o_ = grass reference evaporation (mm day^−1^), = slope of saturation vapor pressure curve (kPa C^−1^), Rn = net radiation (MJ m^−2^ day^−1^), G = soil heat flux density (MJ m^−2^ day^−1^), which was taken as zero for daily estimates, T = daily mean air temperature (C) at 2 m, U2 = mean wind speed at 2 m (m s^−1^), es = saturation vapor pressure (kPa), ea = actual vapor pressure (kPa) and = psychometric constant (0.0677 kPa C^−1^). Co = conversion parameter (= 0.0023).

### Statistical evaluation of models

The empirical model performance can be evaluated using several statistical error metrics. But there is not a single statistic that encompasses all relevant factors. For this reason, it is helpful to take into account a variety of performance data and to comprehend the kind of knowledge or insight they may offer. The open-air package of R software^47^ was used to assess the performance of models. The open air is an R package chiefly developed for the analysis of air pollution measurement data but it is versatile and widely used in the atmospheric and climate sciences^47^. The ranking of model was done with modstat function by considering statistical performance indicators like Fraction of predictions within a factor or two (FAC2), Mean Bias (MB), Mean Gross Error (MGE), Normalized Mean Bias (NMB), Normalized Mean Gross Error (NMGE), Root Mean Square Error (RMSE), correlation coefficient ***r*** and Index of Agreement (IOA). The details of the statistical error metrics are given below. In the following definitions, O_i_ represents the ith observed value and M_i_ represents the ith modelled value for a total of n observations.

### Fraction of predictions within a Factor or two, (FAC2)

The fraction of modelled values within a factor of two of the observed values are the fraction of model predictions that satisfy:

3 $${\text{FAC}}2=0.5 \le \frac{{{\text{M}}}_{{\text{i}}}}{{{\text{O}}}_{{\text{i}}}} \le 2.0$$

### Mean bias (MB)

The mean bias gives a clear indicator of whether model estimates/predicted values are on average over or underestimated. MB expressed in the same units as the variables under consideration^47^.

### Mean gross error (MGE)

This gives mean gross error or simply the mean error without reference to overestimation or underestimation. MGE is expressed in the same units as the input values^47^.

4 $${\text{MB}}= \frac{1}{{\text{n}}}\sum_{{\text{i}}=1}^{{\text{N}}}{{\text{M}}}_{{\text{i}}}-{{\text{O}}}_{{\text{i}}}$$

5 $${\text{MGE}}= \frac{1}{{\text{n}}}\sum_{{\text{i}}=1}^{{\text{N}}}\left|{{\text{M}}}_{{\text{i}}}- {{\text{O}}}_{{\text{i}}}\right|$$

### Normalized mean bias (NMB)

Whether the modeled mean underestimates or overestimates the observed mean is indicated by the sign of NMB. Additionally, the NMB magnitude reveals the degree of under or overestimation^48^.

6 $${\text{NMB}}= \frac{\sum_{{\text{i}}=1}^{{\text{n}}}{{\text{M}}}_{{\text{i}}}-{{\text{O}}}_{{\text{i}}}}{\sum_{{\text{i}}=1}^{{\text{n}}}{{\text{O}}}_{{\text{i}}}}$$

### Normalized mean gross error (NMGE)

The normalized mean gross error further ignores whether a prediction is an over or underestimated.

7 $${\text{NMGE}}= \frac{\sum_{{\text{i}}=1}^{{\text{n}}}\left|{{\text{M}}}_{{\text{i}}}-{{\text{O}}}_{{\text{i}}}\right|}{\sum_{{\text{i}}=1}^{{\text{n}}}{{\text{O}}}_{{\text{i}}}}$$

### Root mean square error (RMSE)

This give the measure of how close modelled values are to predicted values. It ranges from 0 to ∞ and lower the values better the performance

8 $$RMSE= \sqrt{\frac{1}{n}{\sum }_{i=1}^{n}({M}_{i}-{O}_{i}{)}^{2}}$$

### Correlation coefficient (*r*)

Correlation is a measure of the degree of association between two variables. The Pearson's correlation^49^ r is given by

9 $$r= \frac{1}{n-1}\sum_{i=1}^{n}\left(\frac{{M}_{i}-\overline{M}}{\sigma M }\right)\left(\frac{{O}_{i}-\overline{O}}{\sigma O }\right)$$

### Index of agreement (IOA)

The Index of Agreement, IOA compares model estimates or predictions (Pi; I = 1, 2,…, n) with matched pairs of reliable observations (Oi; I = 1, 2,…, n). It spans between −1 and + 1 with values approaching + 1 representing better model performance^50^.

10 $$IOA=\left\{1.0- \frac{\sum_{i=1}^{n}\left|{M}_{i}- {O}_{i}\right|}{c\sum_{i=1}^{n}\left|{O}_{i}- \overline{O }\right|} , when \sum_{i=1}^{n}\begin{array}{c}\left|{M}_{i}- {O}_{i}\right|\le c \sum_{i=1}^{n}\left|{O}_{i}- \overline{O }\right|\\ \end{array}\right.$$

11 $$IOA=\left\{\frac{c \sum_{i=1}^{n}\left|{O}_{i}- \overline{O }\right|}{\sum_{i=1}^{n}\left|{M}_{i}- {O}_{i}\right|}-1, when \sum_{i=1}^{n}\begin{array}{c}\left|{M}_{i}- {O}_{i}\right| >c \sum_{i=1}^{n}\left|{O}_{i}- \overline{O }\right|\\ \end{array}\right.$$

### Taylor diagram

One of the more effective tools for assessing model performance is the Taylor Diagram. The graphic demonstrates the simultaneous variation in three complementing model performance statistics. The correlation coefficient R, standard deviation (sigma), and the (centred) root-mean-square error are these statistics. Due to their relationships with one another, which may be illustrated by the Law of Cosines, these three statistics can be plotted on a single (2D) graph^51^.

## Results and discussion

**Figure 2:**
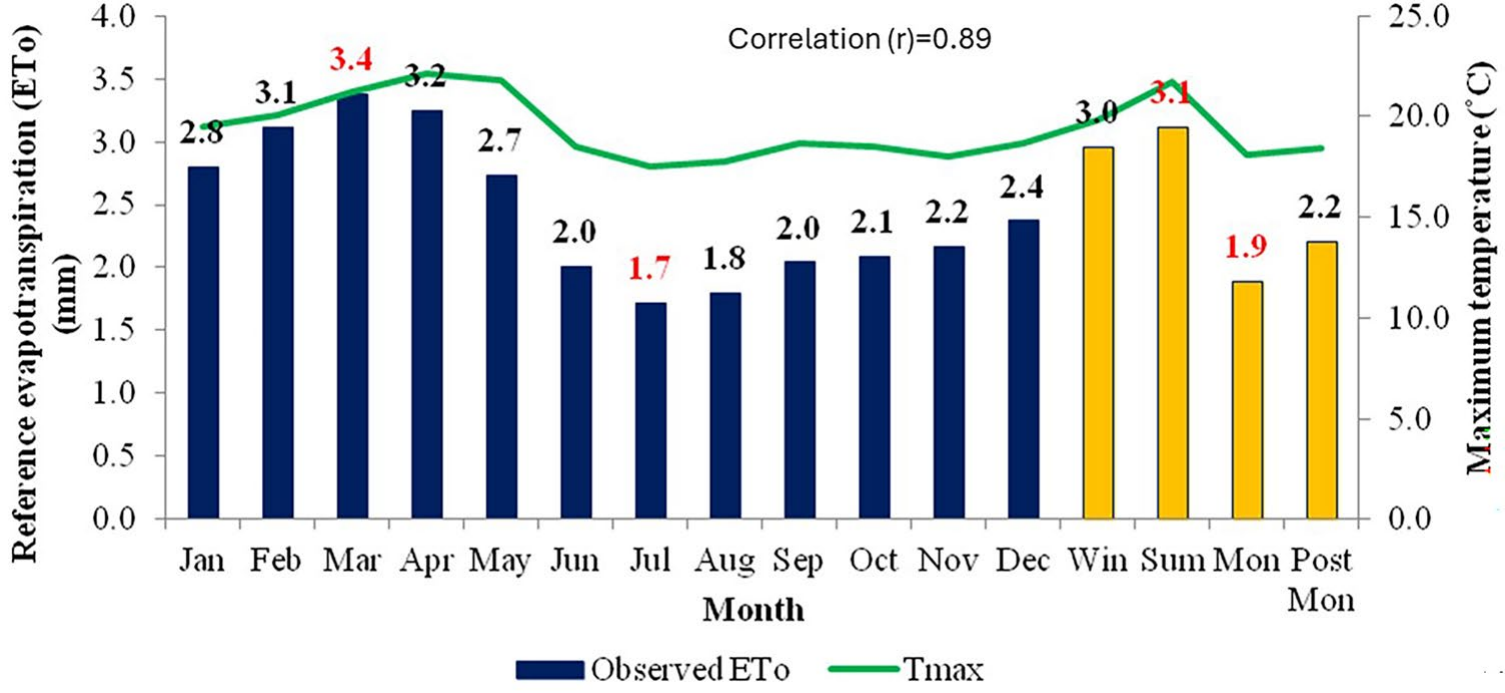
Effect of maximum temperature on average monthly and seasonal pan evaporation for the period 1960–2020.

**Figure 3:**
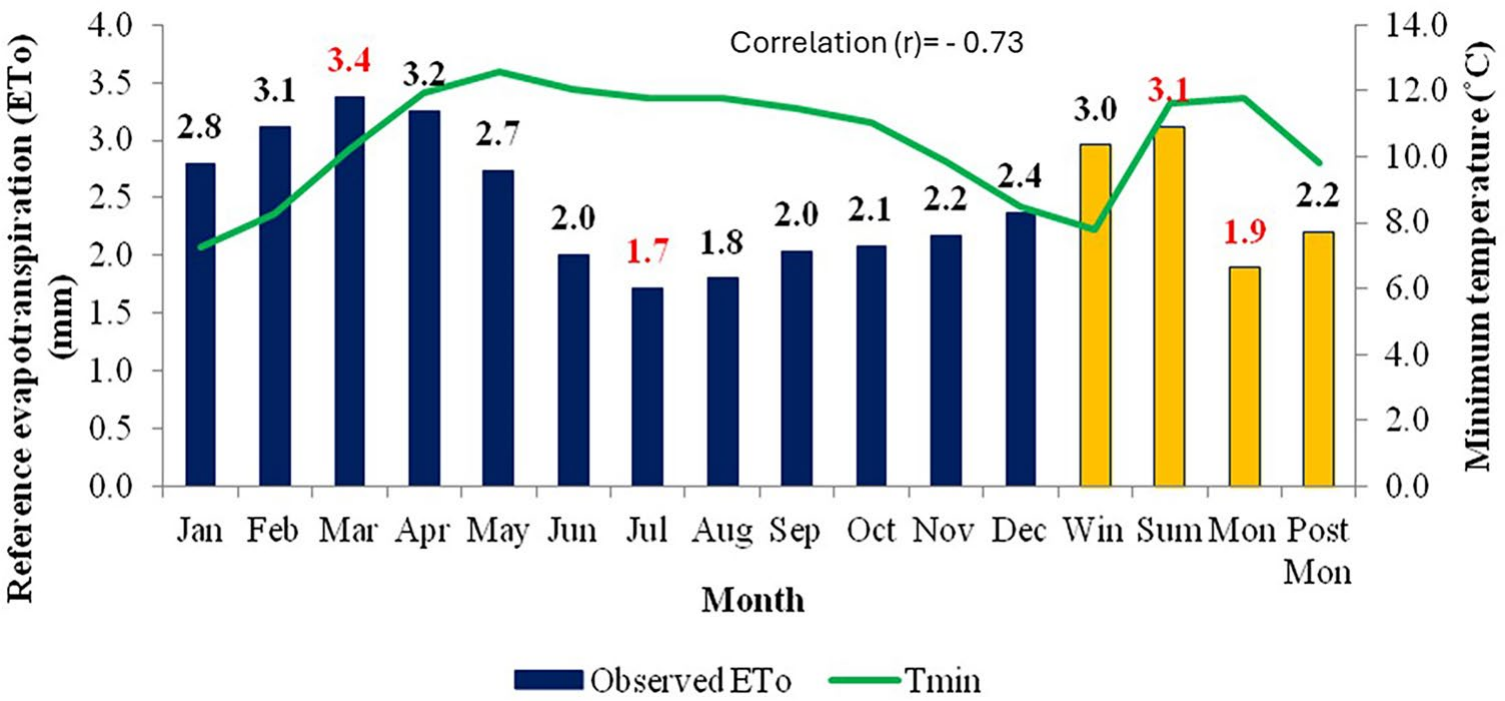
Effect of minimum temperature on average monthly and seasonal pan evaporation for the period 1960–2020.

**Figure 4:**
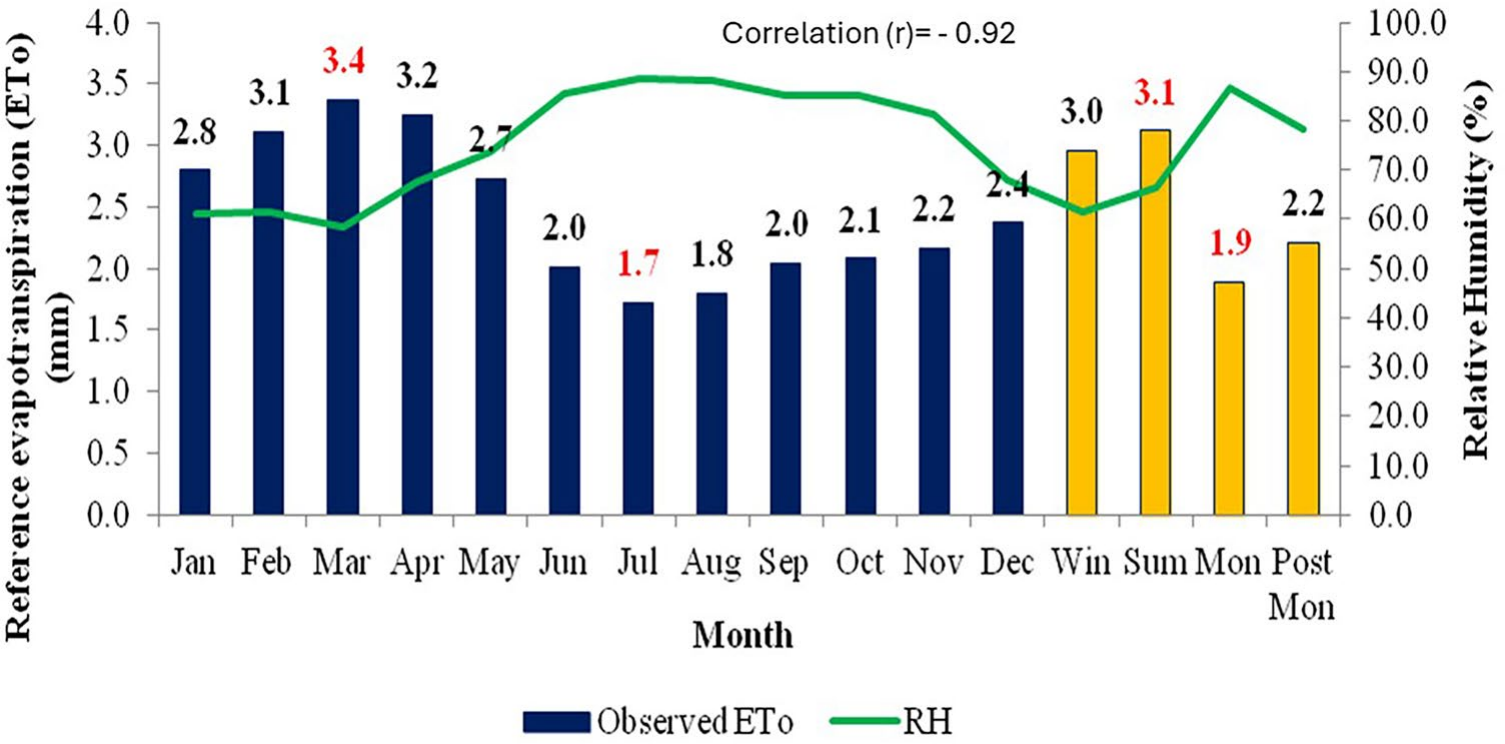
Effect of relative humidity on average monthly and seasonal pan evaporation for the period 1960–2020.

**Figure 5:**
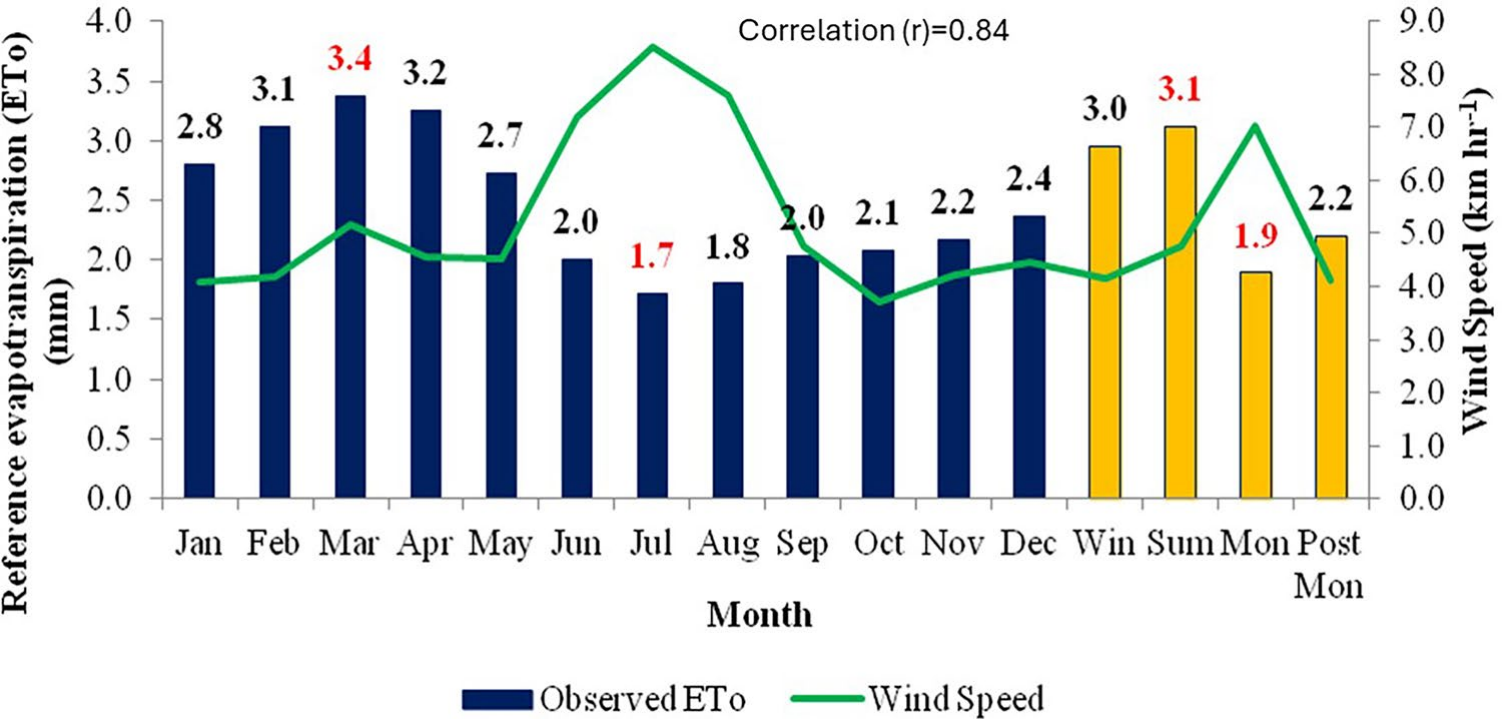
Effect of wind speed on average monthly and seasonal pan evaporation for the period 1960–2020.

**Figure 6:**
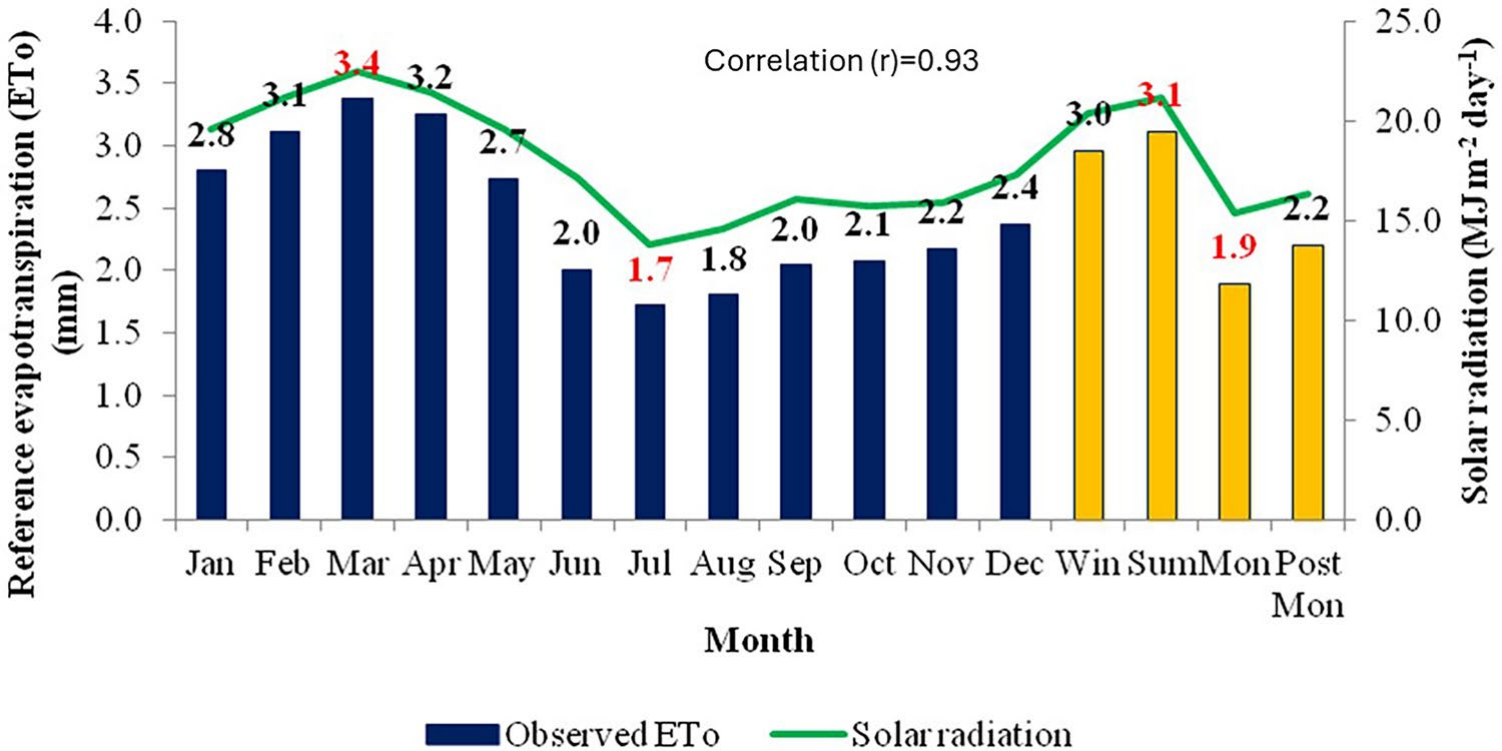
Effect of solar radiation on average monthly and seasonal pan evaporation for the period 1960–2020.

In the study area, the maximum ET_o_ (3.4 mm day^−1^) was observed during March and minimum ET_o_ (1.7 mm day^−1^) was recorded in July. Among the four seasons, maximum ET_o_ was recorded during summer (3.1 mm day^−1^) and minimum during monsoon (1.9 mm day^−1^) as per the water vapour pressure variation in different seasons. These results indicate high water requirements during summer followed by winter and monsoon seasons in the study area. The high-water requirements in summer and winter are due to high air temperatures and low atmospheric humidity in summer and stronger winds in winter which increase the ET_o_ by increased evaporation rate and strong aerodynamic effects respectively. An interesting observation is that the ET_o_ during the monsoon period (June–October) is relatively low compared to other seasons and this period has high humidity (70–90%) due to the study region experiencing strong moist laden southwesterly monsoon winds in this season. The relation between evapotranspiration and meteorological parameters like maximum temperature, minimum temperature, relative humidity, wind speed and solar radiation were plotted in Figs. 2, 3, 4, 5 and 6.

The relationship between ET_o_ and meteorological parameters is fundamental in understanding various aspects of environmental processes, including water availability, ecosystem functioning, and climate dynamics. Mean temperature typically has a direct influence on ET_o_ rates as it enhances the evaporation. As temperatures rise, the energy available for driving the process of evaporation increases. This means that higher temperatures generally lead to higher rates of evapotranspiration. This relationship is evident in regions experiencing warmer climates, where ET tends to be more pronounced, and these Figs. 2, 3, 4, 5 and 6 also confirm the same. Figure 3 shows ET_o_ in different months are inversely correlated with minimum temperatures which is again attributable to temperature effect. It is observed that among the parameters maximum temperature and solar radiation showed a positive correlation with ET_o_, and the other parameters like minimum temperature, relative humidity showed a negative correlation with ET_o_. The correlation values (r)obtained for maximum temperature and solar radiation are 0.89 and 0.93, and in the case of minimum temperature and relative humidity are −0.73 and −0.92, respectively. Regression analysis with these parameters confirmed the same. The moisture abundance in monsoon months also can be seen from relatively high RH in June–October in the study region (Fig. 4). In the case of wind speed, it showed negative relation with evapotranspiration throughout the year except during monsoon, when it showed the increase in wind led to decrease (Fig. 5) in evapotranspiration. The decrease of ET_o_ due to stronger winds during monsoon period is due to increased moisture transport by the stronger monsoon winds (Fig. 5) to the study region which reduces the rate of ET_o_ from plants.

The reference evapotranspiration ET_o_ was calculated using 18 empirical models, which were further grouped into two categories, i.e., Temperature based and radiation-based methods and discussed below.

### Comparison of pan observed and FAO-PM based reference evapotranspiration (ET_o_)

**Figure 7:**
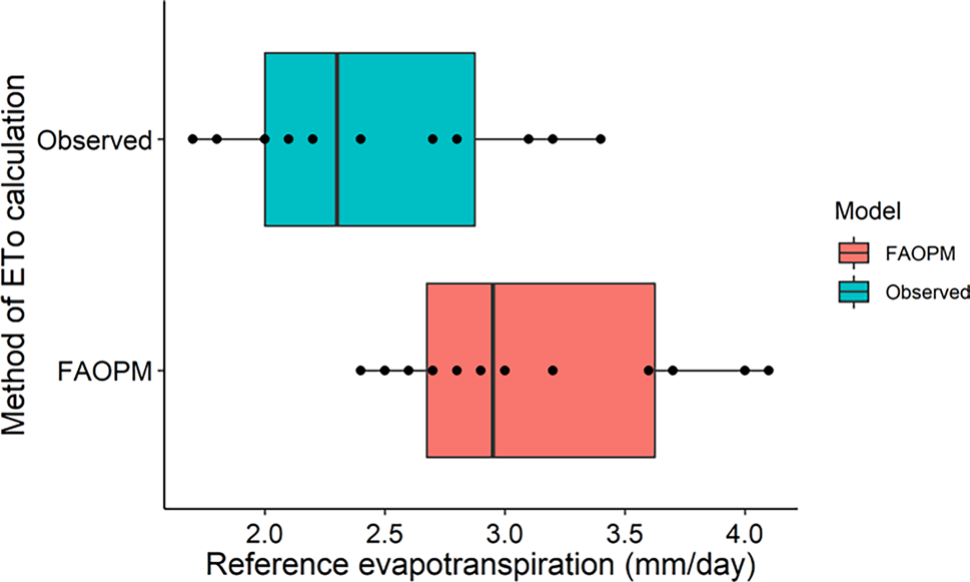
Box plot of FAO-PM based ET_o_ models compared to pan observed ET_o_.

The study compared ET_o_ estimates derived from pan evaporation observation and the FAO Penman–Monteith method (FAO-PM), respectively, which is the internationally accepted method of ET_o_ calculation. The FAO-PM-based ET_o_ ranges from 2.4 to 4.1 mm day^−1^ which is close to the pan observed ET_o_ value, which is 1.7 to 3.4 mm day^−1^ (Table 3, Fig. 7). The FAO-PM method slightly overestimated the pan observed values of ET_o_.

**Table 3:** ET_o_ values (mm) from empirical models.

Month	Jan	Feb	Mar	Apr	May	Jun	Jul	Aug	Sep	Oct	Nov	Dec
	(mm)											
Observed ET_o_	2.8	3.1	3.4	3.2	2.7	2	1.7	1.8	2	2.1	2.2	2.4
FAO-PM	3.2	3.6	4.1	4	3.7	3	2.4	2.5	2.8	2.7	2.6	2.9
Temperature based models												
Hargreaves–Samani	3.2	3.5	3.8	3.9	3.8	2.9	2.7	2.8	3.0	2.9	2.7	2.9
Schendel	3.5	3.7	4.3	4.0	3.7	2.9	2.6	2.7	2.8	2.8	2.7	3.2
Kharrufa	2.6	2.9	3.3	3.8	3.8	3.4	3.2	3.1	3.2	3.0	2.7	2.6
Trajkovic	6.5	7.2	7.8	8.1	7.8	6.3	5.9	6.0	6.4	6.1	5.7	5.9
Berti	2.7	3.0	3.2	3.3	3.2	2.5	2.3	2.4	2.6	2.4	2.3	2.4
Blanney Criddle	3.7	3.9	4.1	4.4	4.5	4.4	4.3	4.1	4.2	4.1	4.0	4.0
Papadakis	1.6	1.7	2.0	1.7	1.3	0.6	0.5	0.5	0.6	0.6	0.8	1.3
Ivanov	3.4	3.5	4.1	3.5	2.8	1.4	1.1	1.1	1.4	1.4	1.7	2.9
Radiation based models												
Makkink	5.0	5.4	5.8	5.5	5.0	4.4	3.5	3.7	4.1	4.0	4.1	4.4
Jensen–Haise	3.3	3.8	4.3	4.4	4.1	3.2	2.5	2.7	3.0	2.9	2.8	3.0
Irmak (Rs)	3.4	3.7	4.0	3.9	3.7	3.1	2.6	2.7	3.0	2.9	2.9	3.0
Irmark (Rn)	3.7	4.1	4.4	4.5	4.3	3.9	3.4	3.5	3.7	3.6	3.4	3.4
Caprio	3.0	3.4	4.0	4.1	3.8	3.0	2.3	2.4	2.8	2.6	2.5	2.7
Jones	3.6	4.0	4.4	4.3	3.9	3.3	2.6	2.7	3.1	3.0	3.0	3.2
Turc	3.2	3.5	3.9	3.9	3.6	3.0	2.4	2.6	2.8	2.7	2.7	2.9
Tabari	2.9	3.2	3.5	3.5	3.3	2.9	2.3	2.5	2.7	2.6	2.5	2.6
Priestley–Taylor	5.4	6.1	6.6	6.7	6.3	5.6	4.8	5.0	5.3	5.1	4.8	4.9
Abtew	2.8	3.1	3.5	3.4	3.1	2.3	1.8	1.9	2.2	2.1	2.1	2.3

### Comparison of pan observed and empirical model-based reference evapotranspiration (ET_o_)

**Figure 8:**
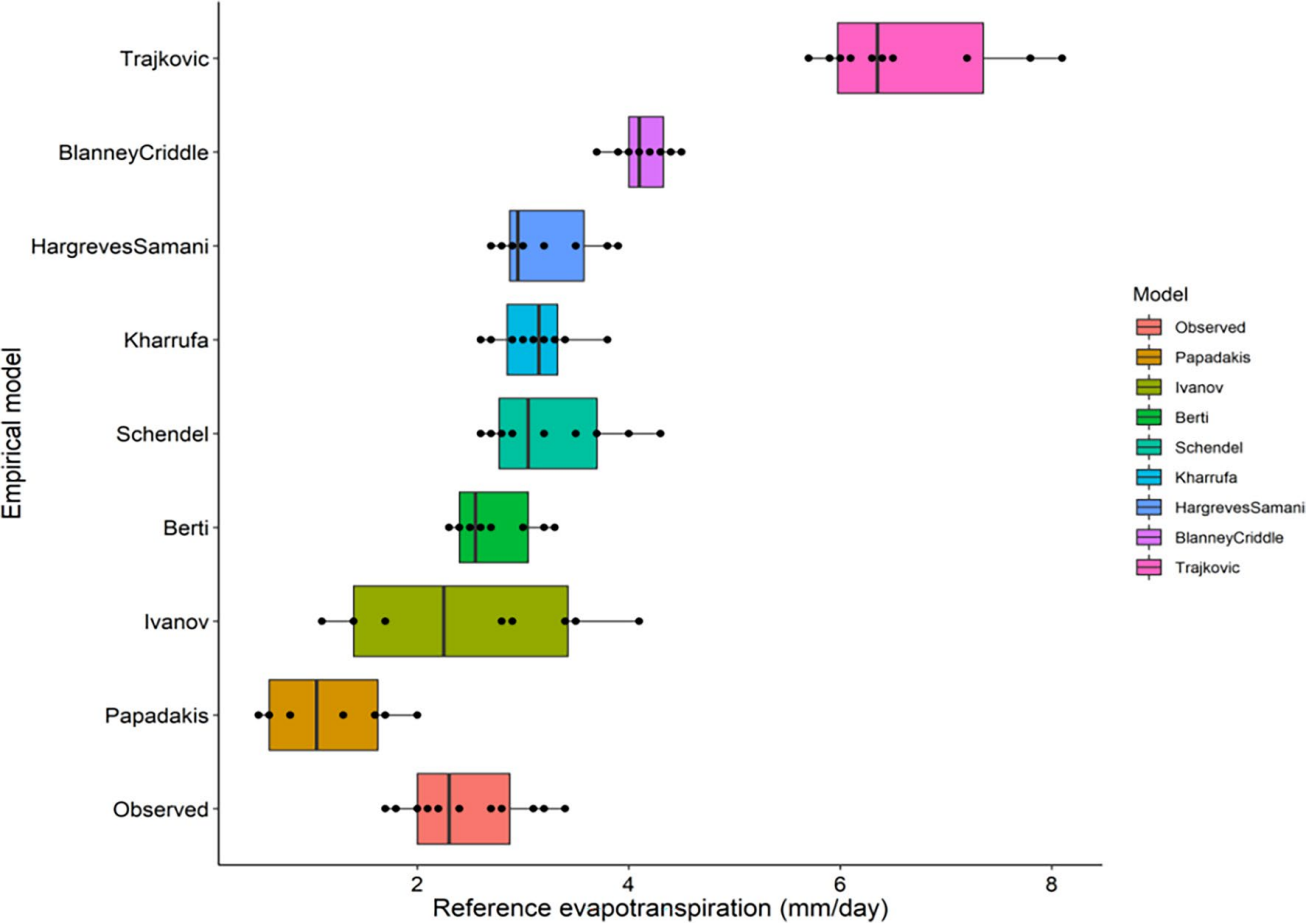
Box plot of temperature based ET_o_ models compared to pan observed ET_o_.

The temperature-based ET_o_ is presented in Table 3 and depicted in a box plot (Fig. 8), along with ET_o_ obtained from pan evaporation observation evapotranspiration. Among the eight temperature-based models assessed, the Papadakis model exhibited an underestimation of ET_o_ ranging from 0.5 to 2 mm day^−1^, while the Trajkovic method showed an overestimation of ET_o_ ranging from 5.7 to 8.1 mm day^−1^. This discrepancy in the Papadakis model can be attributed to its reliance solely on water vapor pressure deficit, while the overestimation in the Trajkovic method results from its consideration of both radiation and temperature without factoring in the role of atmospheric humidity. The vapor pressure deficit-based relation of ET_o_ is effective primarily under dry conditions but may prove inadequate under humid conditions.

The Ivanov model reported the widest range of evapotranspiration with the lowest value as 1.1 mm day^−1^ and highest as 4.1 mm day^−1^, whereas Blaney Criddle model has the lowest ET_o_ range from 3.7 to 4.5 mm day^−1^. The Berti model which is based on both net radiation and maximum, minimum and mean temperatures gave an ET_o_ value (2.3 to 3.3 mm day^−1^) close to the pan observed value. The Schendel (2.7 to 4.3 mm day^−1^), Hargreaves–Samani (2.7 to 3.9 mm day^−1^) and Kharrufa (2.6 to 3.8 mm day^−1^) methods also gave a similar range of ET_o_.

**Figure 9:**
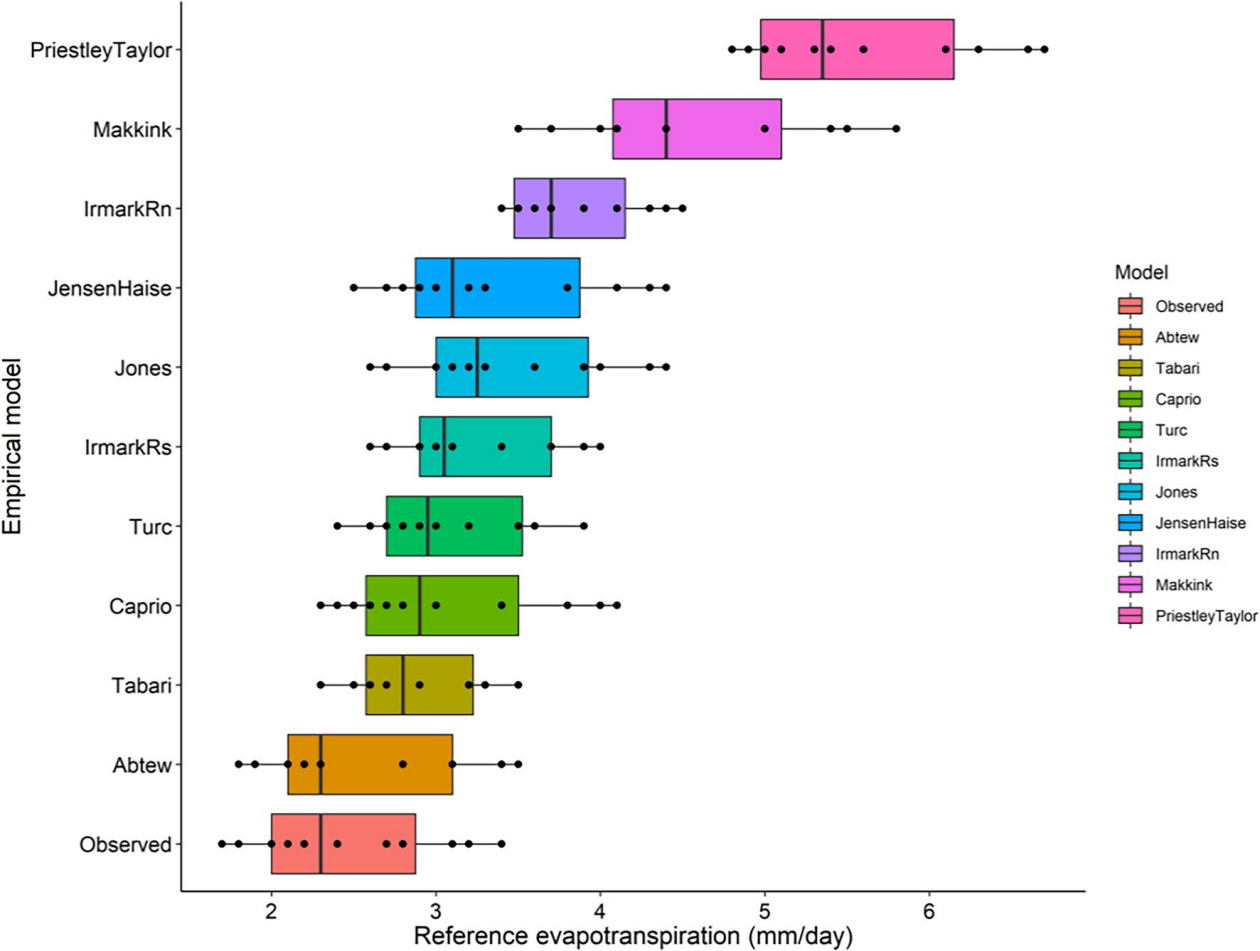
Box plot of radiation based ET_o_ models compared to pan observed ET_o_.

In the case of radiation-based ET_o_ methods (Table 3, Fig. 9), the Abtew method showed the lowest ET_o_ values (1.8 to 3.5 mm day^−1^) which were close to observed values (1.7 to 3.4 mm day^−1^). Tabari (2.3 to 3.5 mm day^−1^), Caprio (2.3 to 4.1 mm day^−1^) and Turc (2.4 to 3.9 mm day^−1^) methods also showed a similar range of ET_o_. The range of ET_o_ was nearly similar in Irmark (Rs) (2.6 to 3.9 mm day^−1^), Jensen–Haise (2.5 to 4.4 mm day^−1^) and Jones (2.6 to 4.4 mm day^−1^) methods of calculation. The highest range of ET_o_ was observed in Irmark (Rn) (3.4 to 4.5 mm day^−1^), Makkink (3.5 to 5.8 mm day^−1^) and Priestley Taylor (4.8 to 6.7 mm day^−1^) methods.

### Comparison of FAO-PM and empirical models based reference evapotranspiration (ET_o_)

**Figure 10:**
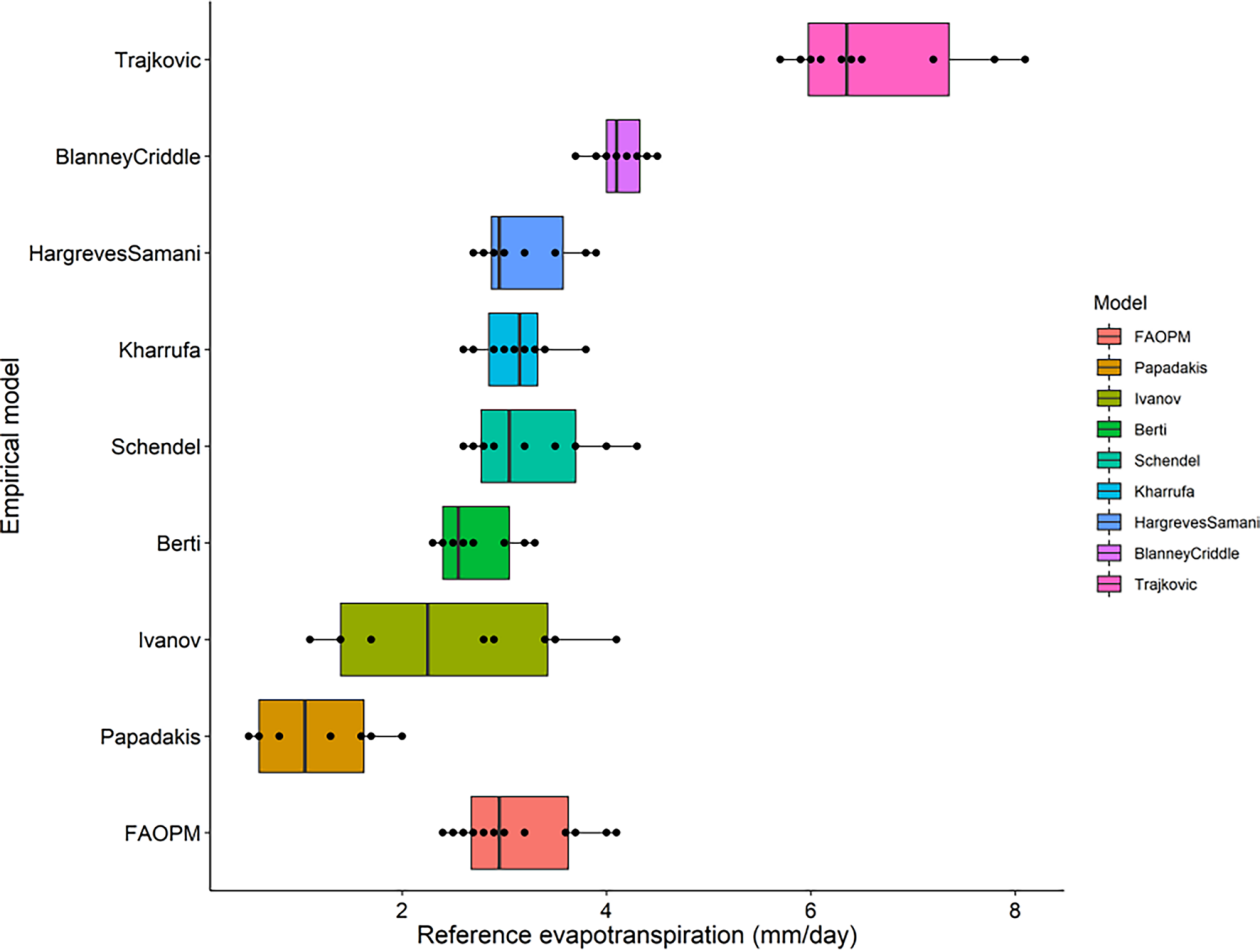
Box plot of temperature based ET_o_ models compared to FAO-PM based ET_o_.

**Figure 11:**
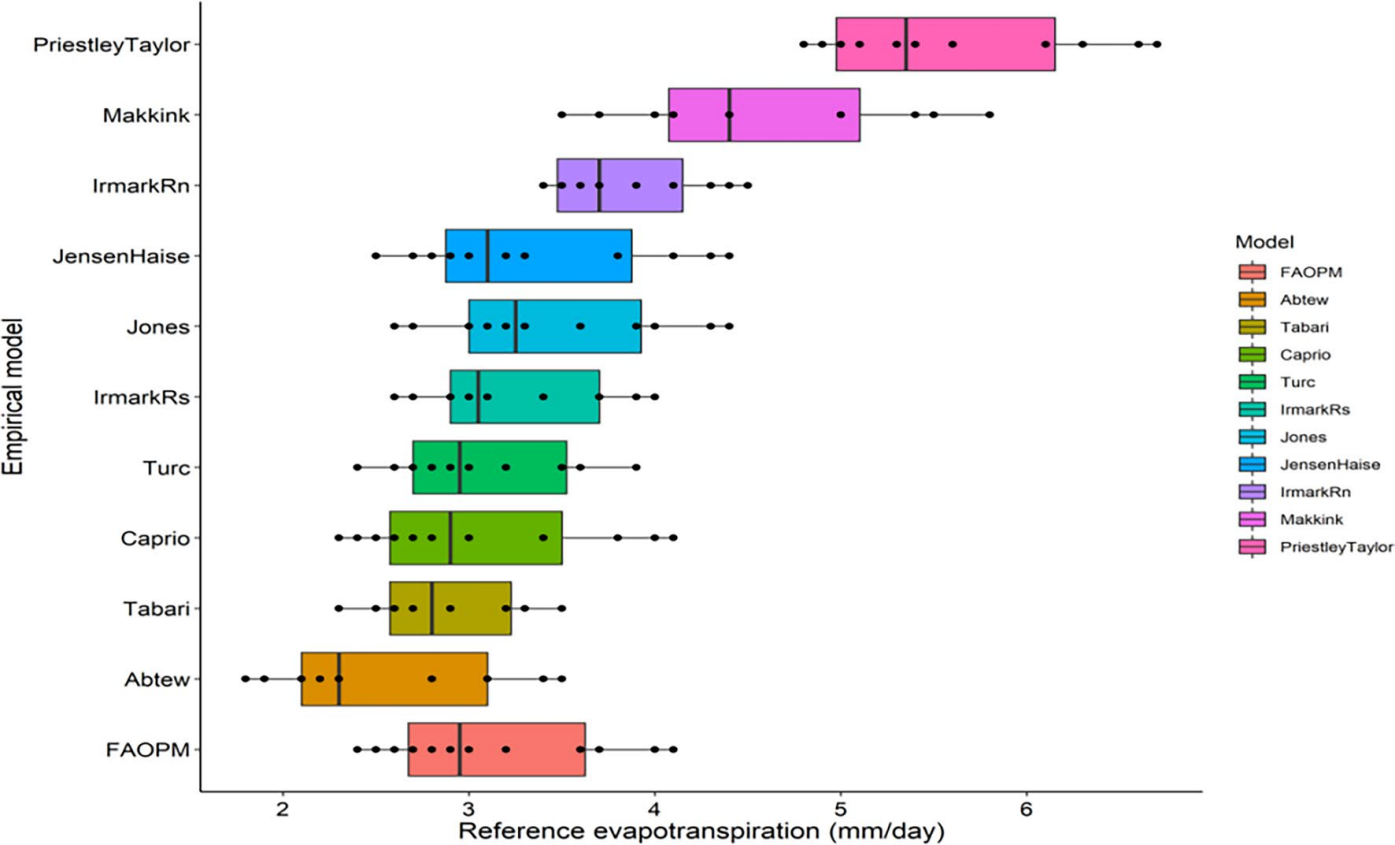
Box plot of radiation based ET_o_ models compared to FAO-PM based ET_o_.

The temperature-based ET_o_ calculation was compared with the FAO-PM method and plotted in Fig. 10. It was observed that Papadakis method underestimated the FAO-PM values, whereas Trajkovic and Blaney Criddle's methods overestimated the same. The comparison of radiation-based models and FAO-PM was depicted in Fig. 11. The Makkink and Priestley–Taylor models overestimated the ET_o_, whereas all other models were close to the FAO-PM values.

### Statistical performance of FAO-PM model with pan observed ET_o_

The comparison of FAO-PM based ET_o_ with with ET_o_ obtained from pan evaporation observation, showed good performance in terms of all the statistical indices considered (Table 4). The high values of FAC2 (1) and r (0.95), and low values of MB (0.66), MGE (0.66), NMB (0.27) and NMGE (0.27) indicate good performance of the FAO-PM model. Study of Vishwakarma et al.^52^ compared ET_o_ obtained from pan evaporation observation and with ET_o_ from FAO PM method for humid and subtropical conditions of Ludhiana district of Punjab, India and observed better performance of the FAO-PM method with good RMSE value of 1.13 and r value of 0.96. The radiation-based models also consider temperature variables compared to the temperature-based models as ET_o_ depends on Rs (available energy for evaporation) as well as the temperature of the air.

**Table 4:** Statistical performance of empirical models with respect to ET_o_ estimates derived from pan evaporimeter observations.

Models	FAC	MB	MGE	NMB	NMGE	RMSE	r	IOA
FAO-PM	1	0.66566	0.66566	0.271218	0.271218	0.689075	0.947607	0.332
Temperature based models								
Berti	1.00	0.22	0.29	0.09	0.12	0.35	0.90	0.71
Ivanov	1.00	−0.09	0.52	−0.04	0.21	0.56	0.96	0.47
Hargreaves–Samani	1.00	0.73	0.73	0.30	0.30	0.77	0.90	0.27
Kharrufa	1.00	0.70	0.78	0.28	0.32	0.93	0.18	0.21
Schendel	1.00	0.79	0.79	0.32	0.32	0.80	0.97	0.20
Papadakis	0.42	−1.36	1.36	−0.55	0.55	1.36	0.97	−0.26
Blaney Criddle	0.67	1.69	1.69	0.69	0.69	1.80	−0.12	−0.41
Trajkovic	0.00	4.17	4.17	1.70	1.70	4.19	0.85	−0.76
Radiation based models								
Abtew	1.00	0.10	0.12	0.04	0.05	0.16	0.97	0.88
Tabari	1.00	0.42	0.42	0.17	0.17	0.48	0.93	0.58
Caprio	1.00	0.61	0.61	0.25	0.25	0.66	0.90	0.39
Turc	1.00	0.65	0.65	0.26	0.26	0.67	0.95	0.35
Irmark(Rs)	1.00	0.78	0.78	0.32	0.32	0.80	0.96	0.22
Jensen–Haise	1.00	0.87	0.87	0.36	0.36	0.91	0.91	0.13
Jones	1.00	0.97	0.97	0.39	0.39	0.98	0.97	0.03
Irmark(Rn)	1.00	1.35	1.35	0.55	0.55	1.39	0.83	−0.26
Makkink	0.67	2.12	2.12	0.86	0.86	2.13	0.98	−0.53
Priestley–Taylor	0.25	3.10	3.10	1.26	1.26	3.12	0.85	−0.68

### Statistical performance of empirical models with Pan observed ET_o_

**Figure 12:**
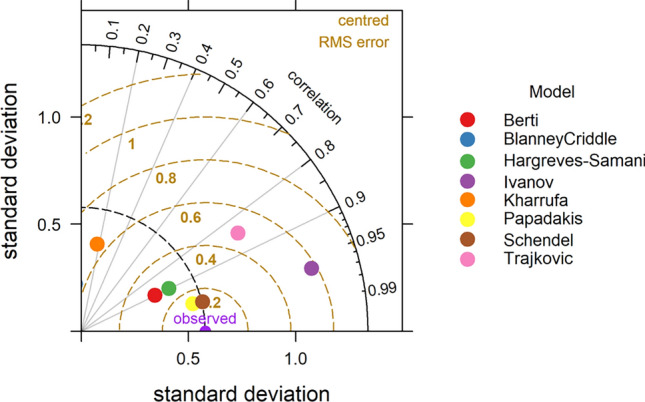
Taylor diagram depicting relation between pan observed and temperature based ET Models.

The statistical analysis of temperature-based models is presented in Table 4. It is observed that all the models gave FAC2 value of 1 except Papadakis (0.42), Blaney Criddle (0.67) and Trajkovic (0.0), which indicate better performance of most of the models. Overall, the Berti model was found to be the best performing model with MB = 0.22, MGE = 0.29, NMB = 0.09, NMGE = 0.12, RMSE = 0.35, r = 0.9 and IOA = 0.71. The Ivanov model also gave better results with FAC2, MB, MGE, NMB, NMGE, RMSE, r and IOA values respectively. The poor performed models were Blaney Criddle and Trajkovic, where Blaney Criddle gave FAC2 value of 0.67, MB of 1.69, MGE of 1.69, NMB of 0.69, NMGE of 0.69, RMSE of 1.8, r value of −0.12 and IOA value of −0.41. The negative ‘r’ value in Blaney Criddle method indicates a negative linear relationship with observed values. In the case of the Trajkovic method the FAC2, MB, MGE, NMB, NMGE, RMSE, r and IOA values are 0.00, 4.17, 4.17, 1.7, 1.7, 4.19, 0.85 and −0.76 respectively. The negative values of MB and NMB IN Ivanov (MB = −0.09, NMB = −0.04) and Papadakis (MB = −1.36, NMB = −0.55) indicate underestimation of ET_o_ values in these methods. The Taylor diagram indicating the performance of the models is presented in the Fig. 12. The relative performance of different tested models is as follows Beri > Ivanov > Hargreves > Kharrufa > Schendel > Papadakis > Blaney Criddle > Trajkovic.

**Figure 13:**
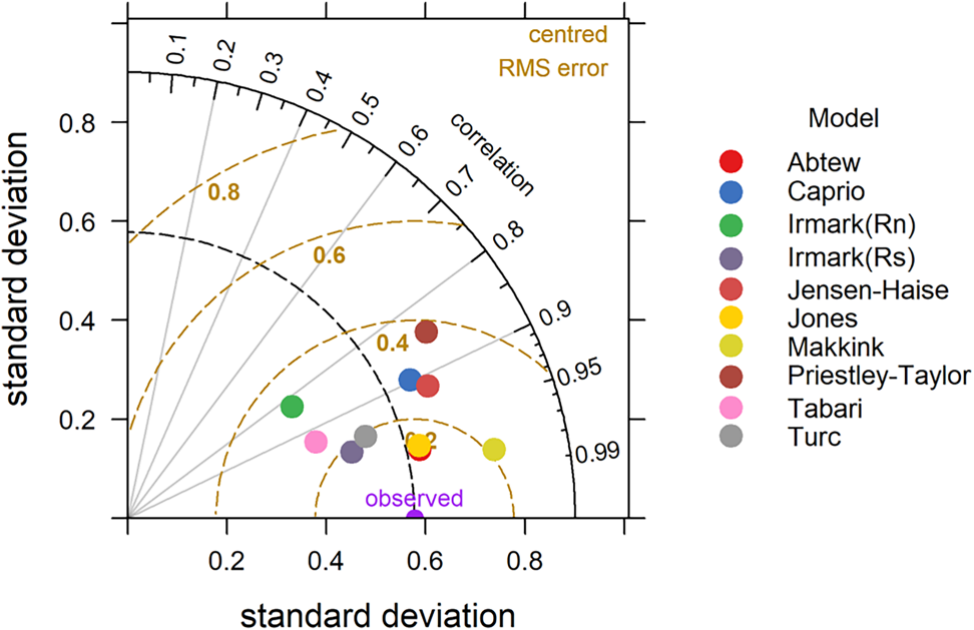
Taylor diagram depicting relation between pan observed and radiation based ET_o_ Models.

In case of radiation-based models (Table 4), The Abtew and Tabari methods performed better with respect to pan observed ET_o_. In Abtew method gave FAC2 = 1, MB = 0.10, MGE = 0.12, NMB = 0.04, NMGE = 0.05, RMSE = 0.16, r = 0.97 and IOA = 0.88 values, which displays better performance of the model. For the Tabari model also the statistical performance was better with FAC2 = 1, MB = 0.42, MGE = 0.42, NMB = 0.17, NMGE = 0.17, RMSE = 0.48, r = 0.93 and IOA = 0.58 values. Even though the Makkink method yield high value of r (0.98), the other statistical indicators like FAC2 (0.67), MB (2.12), MGE (2.12) NMB (0.86), NMGE (0.86), RMSE (2.13) and IOA (−0.53) gave poor values, resulted in the worst performance of the model. The Priestley Taylor method gave the highest value of MB (3.10) and NMB (1.26) indicating highest over estimation. The overall performance of the model was worst with poor values of FAC2 (0.25), MGE (3.10), NMGE (1.26), RMSE (3.12), r (0.85) and IOA (−0.68). The performance of the models as given by the Taylor diagram is shown in Fig. 13. Among all the radiation-based models, Abtew performed best, followed by Jones, Tabari, Caprio, Turc, Irmark (Rs), Jensen–Haise, Irmark (Rn), Makkink and Priestley Taylor.

### Statistical performance of empirical models with FAO-PM ET_o_

**Figure 14:**
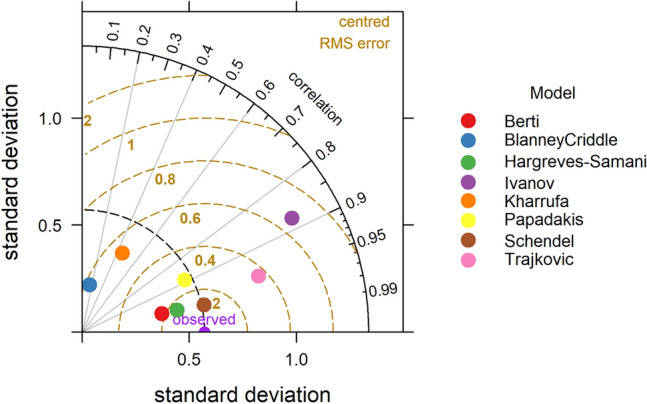
Taylor diagram depicting relation between FAO-PM and temperature based ET_o_ Models.

The statistical performance of temperature-based models with respect to the FAO-PM method differs slightly from what was observed with respect to pan observed ET_o_ (Table 5). The Hargreaves model recorded best performance with FAC2 = 1, MB = 0.07, MGE = 0.13, NMB = 0.02, RMSE = 0.17, r = 0.97 and IOA = 0.87. The superior performance of the Hargreaves–Samani model was reported by Bogawski and Bednorz^53^ under the central European condition. They reported that when only temperature or pan evaporation data were available, the calibrated Hargreaves–Samani method provided the most accurate results (RE = 0.275). The better performance of Hargreaves ET_o_ model was also reported by Mandal et al.^15^ for different stations in India. The Schendel method also produced similar results with FAC2, MB, MGE, NMB, NMGE, RMSE, r and IOA values as 1, 0.13, 0.15, 0.04, 0.05, 0.18, 0.98 and 0.85 respectively. The Trajkovic (FAC2 = 0.08, MB = 3.50, MGE = 3.5, NMB = 1.12, NMGE = 1.12, RMSE = 3.52, r = 0.95 and IOA = −0.72) and Papadakis (FAC2 = 0.00, MB = −2.02, MGE = 2.02, NMB = −0.65, NMGE = 0.65, RMSE = 2.04, r = 0.89 and IOA = −0.52) performed worst among the models. The negative MB and NMB values of Berti, Ivanov and Papadakis models indicates the underestimation of ET_o_ in these models, when compared to FAO-PM method. The overall performance of temperature-based models are in the order of Hargreaves–Samani > Schendel > Berti > Kharrufa > Ivanov > Blaney- Criddle > Papadakis > Trajkovic. The performance of the models is depicted in Taylor diagram Fig. 14.

**Table 5:** Statistical performance of empirical models with respect to estimated ET_o_ using FAO-PM.

Models	FAC	MB	MGE	NMB	NMGE	RMSE	r	IOA
Temperature based models								
Hargreaves–Samani	1.00	0.07	0.13	0.02	0.04	0.17	0.97	0.87
Schendel	1.00	0.13	0.15	0.04	0.05	0.18	0.98	0.85
Berti	1.00	−0.45	0.45	−0.14	0.14	0.49	0.97	0.54
Kharrufa	1.00	0.03	0.45	0.01	0.14	0.51	0.45	0.54
Ivanov	0.75	−0.76	0.81	−0.24	0.26	0.99	0.88	0.17
Blaney-Criddle	1.00	1.02	1.02	0.33	0.33	1.16	0.15	−0.06
Papadakis	0.00	−2.02	2.02	−0.65	0.65	2.04	0.89	−0.52
Trajkovic	0.08	3.50	3.50	1.12	1.12	3.52	0.95	−0.72
Radiation based models								
Turc	1.00	−0.02	0.06	−0.01	0.02	0.07	1.00	0.94
Caprio	1.00	−0.06	0.11	−0.02	0.03	0.12	0.99	0.89
Irmark(Rs)	1.00	0.12	0.14	0.04	0.05	0.16	1.00	0.85
Jensen–Haise	1.00	0.21	0.21	0.07	0.07	0.23	0.99	0.79
Tabari	1.00	−0.25	0.25	−0.08	0.08	0.30	0.99	0.74
Jones	1.00	0.30	0.30	0.10	0.10	0.31	0.99	0.69
Abtew	1.00	−0.57	0.57	−0.18	0.18	0.57	0.99	0.41
Irmark(Rn)	1.00	0.68	0.68	0.22	0.22	0.72	0.95	0.29
Makkink	1.00	1.45	1.45	0.47	0.47	1.47	0.97	−0.34
Priestley Taylor	1.00	2.43	2.43	0.78	0.78	2.44	0.96	−0.60

**Figure 15:**
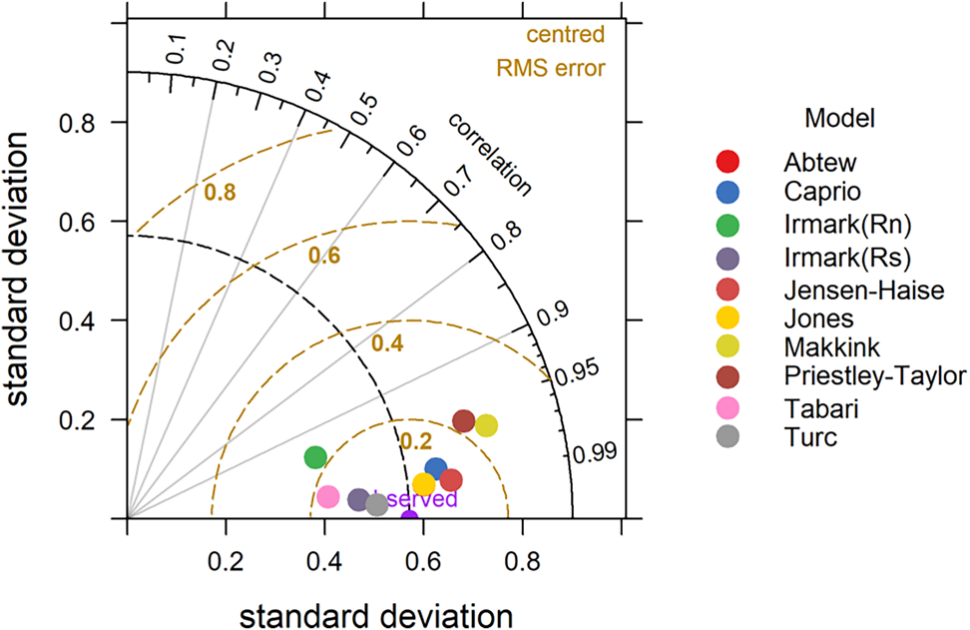
Taylor diagram depicting relation between FAO-PM and radiation based ET_o_ Models.

In the case of radiation based models the Turc method yielded better performance with least bias and error values (MB = −0.02, NMB = −0.01, MGE = 0.06, NMGE = 0.02, RMSE = 0.07) (Table 4). Highest values of FAC2 (1), r (1.00) and IOA (0.94) are also observed in this model. According to Tabari^54^, the Turc model produced the best ET_o_ values for the cold humid climatic condition of Iran. The model was first created for humid environments, which is most likely why it performs best in humid climates. Additionally, Trajkovic and Kolakovic^55^ discovered that this model may deliver accurate ET_o_ estimations in humid climates. The second-best method was found to be Caprio with FAC2, MB, MGE, NMB, NMGE, RMSE, r and IOA values as 1, −0.06, 0.11, −0.02, 0.07, 1.00 and 0.94 respectively. The negative values of MB and NMB in Turc, Caprio, Tabari and Abtew are denoting the under estimation of ET_o_ values in these models. The poor performance was observed in Priestley–Taylor (MB = 2.43, MGE = 2.43, NMB = 0.78, NMGE = 0.78, RMSE = 2.44, r = 0.96, IOA = −0.60) and Makkink (MB = 1.45, MGE = 1.45, NMB = 0.47, NMGE = 0.47, RMSE = 1.47, r = 0.97, IOA = −0.34) model. Tabari reported the worst performance of the Priestley Taylor model in the most humid regions of Iran^16^ (Table 5). From the Taylor diagram (Fig. 15) the performance of different radiation-based models is Turc, followed by Caprio, Irmark (Rs), Jensen–Haise, Tabari, Jones, Abtew, Irmark (Rn), Makkink and Priestley–Taylor.

The comparison study of various models against pan observations and the FAO PM methods across all seasons, showed that radiation-based models exhibited superior performance over temperature-based ones. This superiority can be attributed to the significant influence of Rs (solar radiation) in driving ET_o_ in tropical humid climates^41,56^. Rs plays a pivotal role in redistributing surface energy into various forms (latent, sensible, and ground heat fluxes), thereby facilitating moisture transport through evaporation/evapotranspiration from vegetated surfaces. The reliance of radiation-based models on net radiation to estimate ET_o_ values^35,40,57^ underscores the crucial role of net radiation in governing surface energy balance. Consequently, radiation-based models offer reliable estimates of ET_o_ values. However, it is noteworthy that both radiation and temperature-based empirical models demonstrated suitability for the study region, which experiences high altitudes and maintains moderate to high humidity levels throughout the year. The effectiveness of both types of models in this region can be attributed to the limited influence of atmospheric moisture in regulating ET_o_^56–58^.

## Conclusion

The present study evaluated the performance of different empirical ET_o_ models for the temperate hilly region of Udhagamandalam in South India based on long-term meteorological data. From a detailed comparison of different empirical ET_o_ models with pan observed and FAO PM methods considering the entire year covering all the seasons, it is found that the radiation-based models performed better than the temperature based models. Overall, the radiation-based models outperformed the other empirical models due to the more significant role of solar radiation (Rs) in driving ET_o_ in tropical humid climates. This shows that Rs is the controlling factor in the partitioning of surface energy to different forms (latent, sensible, and ground heat fluxes) which leads to transport of moisture through evaporation/evapotranspiration from vegetated surface. These results confirm that the radiation-based models can reliably estimate ET_o_ values possibly due to net radiation largely determining the evaporation requirements as it effectively controls the surface energy balance. However, in the case of non-availability of radiation data, temperature based empirical models can also be used for the study region. The findings from the study suggests that simple temperature based empirical models can be used for estimation of ET_o_ and that will help in devising suitable irrigation management decisions such as crop water requirement, irrigation scheduling and related agricultural water management practices.

## Data Availability

All data generated or analysed during this study are included in this published article. The datasets (Raw data) used and/or analyzed during the current study available from the corresponding author on request.
